# Weight maintenance interventions for people with type 2 diabetes mellitus: a systematic review protocol

**DOI:** 10.1186/s13643-020-01467-7

**Published:** 2020-09-12

**Authors:** Sarah Sauchelli, Julia Bradley, Jennifer Cox, Clare England, Rachel Perry

**Affiliations:** National Institute for Health Research Bristol Biomedical Research Centre, University Hospitals of Bristol and Weston NHS Foundation Trust and University of Bristol, Level 3, University Hospitals Bristol Education and Research Centre, Upper Maudlin Street, Bristol, BS2 8AE UK

**Keywords:** Weight maintenance, Type 2 diabetes, Systematic review

## Abstract

**Background:**

Weight loss maintenance is a challenge for people with type 2 diabetes mellitus (T2DM), which attenuates the long-term benefits of weight loss for diabetes management. Medication, specific dietary requirements and the psychosocial burden of T2DM signify that weight loss maintenance designed for obesity may not suit people with T2DM. The primary objective of this review is to comprehensively evaluate existing weight maintenance interventions for people with or at high risk of T2DM.

**Methods:**

We registered a protocol for the systematic review and meta-analysis of randomised and non-randomised weight maintenance interventions for T2DM. Studies included will have been carried out in adults with clinical diagnosis of T2DM or pre-diabetes. All intervention types will be accepted (e.g*.* behavioural/lifestyle change and pharmacological). The primary outcomes will be weight control, glycaemic control and adverse effects. Secondary outcomes will include cardiovascular risk factors (e.g. total cholesterol, LDL-cholesterol, blood pressure control), psychological wellbeing (including health-related quality of life), change in glucose medication and waist circumference. Multiple electronic databases will be searched such as MEDLINE, EMBASE, Web of Science, PsychINFO and international registers (e.g. Cochrane Central Register of Controlled Trials, WHO ICTRP). OpenGrey will be searched for grey literature. Two researchers will screen all citations and abstracts. This process will also be conducted by an additional researcher using a semi-automated tool to reduce human error. Full-text articles will be further examined by the researchers to select a final set for further analysis, and a narrative synthesis of the evidence will be presented. Potential sources of heterogeneity will be assessed, and a meta-analysis will be conducted if feasible. Risk of bias will be evaluated using the Cochrane risk of bias tool and the certainty of evidence using the GRADE (grading of recommendations, assessment, development and evaluation) approach.

**Discussion:**

This review will critically appraise existing weight maintenance interventions targeting T2DM. Findings will inform future intervention development to support people with T2DM delay weight regain and prolong successful diabetes management.

**Systematic review registration:**

PROSPERO CRD42020168032

## Background

The International Diabetes Federation reports that in 2019 approximately 463 million adults between ages 20 and 79 were living with diabetes mellitus, with projections estimating a rise to 700 million by 2045 [1]. The existence of multiple comorbidities and an augmented risk of mortality have placed diabetes mellitus as a global health and development challenge [1]. More than 90% of people with diabetes mellitus have type 2 diabetes mellitus (T2DM). Although a genetic predisposition results in an increased susceptibility among some individuals, excess weight, due to poor diet and insufficient physical activity, is the key modifiable risk factor [2]. Hence, lifestyle change to maintain a healthy weight is a fundamental component of existing prevention and management strategies [3].

Extensive literature shows that lifestyle interventions, particularly those focusing on diet control, enable weight loss, ameliorate cardiometabolic health and HbA1c control [4–7]. More recently, it has been demonstrated that a very low-calorie diet encouraging weight loss enables some people with T2DM reach a remission-like state [8]. However, the majority of people with T2DM struggle to maintain the weight lost once weight loss interventions have ended [9]. At 12-month follow-up, the intervention benefits for weight, HbA1c levels, lipids and blood pressure are less clear [10]. There is also some evidence that weight loss maintenance is short-lived in people with T2DM compared to non-diabetic people with obesity [11] or people at high risk [6].

The primary target for diabetes management is glucose control [3] and commonly used pharmacological treatments for glucose regulation facilitate weight gain [3, 12]. Medication used to treat psychiatric comorbidities and diabetes-related medical complications can have a similar side effect [13, 14]. The specific dietary requirements and the psychosocial burden of diabetes self-management can also influence adherence to physical activity and dietary guidelines among this clinical group [15]. Behavioural interventions shown to be beneficial for weight loss maintenance in obesity [16, 17] may therefore not be equally applicable to people who additionally have T2DM.

The prescription of weight maintenance programmes is among the American Diabetes Association’s recommendations for medical care in diabetes [18]. Further, 4-year weight maintenance (< 5% change from baseline measure), particularly when combined with glycaemic control, has been found to result in a reduction in medical costs, compared to a 14% increase when weight gain occurs alongside an incremental increase in mean A1C levels [19]. Given the importance of weight maintenance for diabetes-related health outcomes and the economic savings for the healthcare system, development and implementation of weight maintenance interventions that are effective for people with or at high risk of T2DM is crucial. Comprehensive reviews on intervention effectiveness are fundamental for evidence-based decision-making in clinical practice [20]. Yet, interventions focusing on weight maintenance for people with T2DM are yet to be systematically summarised and assessed for quality.

## Aims

The systematic review described in this protocol aims to thoroughly evaluate the effectiveness of existing weight maintenance interventions that have targeted people with or at high risk of T2DM. It will consider glucose regulation and weight as primary outcomes, in order to discern features of current interventions that may aid patients adhere to treatment targets and to identify challenges to be addressed by novel interventions.

A second objective of this review is to further evaluate the applicability of text-mining tools and machine learning to support clinical systematic reviews. As the rate of publication rises across disciplines, evidence synthesis is becoming increasingly more time-consuming [21]. Automated tools/processes may reduce workload for researchers. However, the use of varied terminology to define medical phenomena and clinical interventions often requires complex decision-making that may be difficult to predict prior to screening. Therefore, the traditional systematic review screening process will be carried out in parallel to a protocol using automated approaches to identify where human judgement may be needed when conducting systematic reviews of studies evaluating clinical interventions.

## Methods

The present study protocol is reported in accordance with the reporting guidance provided in the Preferred Reporting Items for Systematic Reviews and Meta-Analyses Protocols (PRISMA-P) statement [22] (Additional file 1). This protocol has been registered in the International Prospective Register of Systematic Reviews (PROSPERO) database (registration ID: CRD42020168032).

### Eligibility criteria

Both published and unpublished studies including a weight maintenance intervention will be analysed. Studies will be included irrespective of the country where they have been carried out, year of publication and the language of the report. Studies may be both randomised and non-randomised, and blinding is not required given that it is not always possible in behavioural interventions. The use of the findings from this review to develop a novel intervention and design a randomised controlled trial justify the inclusion of non-randomised studies [23]. Studies meeting the following criteria will be deemed eligible for analysis (PICO structure).

#### Population

Participants will be adults (age ≥ 18 years), with a clinical diagnosis of T2DM or pre-diabetes according to the criteria of the World Health Organisation [24] or equivalent international standards [25]. When it is unclear whether a diagnosis was conducted, the study authors will be contacted to obtain the information. Further, studies where participants display impaired fasting glucose or non-diabetic hyperglycaemia will also be included. Participants will additionally have had to achieve weight loss within 24 months prior to the start of the weight maintenance intervention or have been instructed to maintain their weight as a preventative measure. No restriction will be placed on the amount of weight loss achieved during a weight loss programme given differential targets and success rates across studies [26]. Participants may have been recruited from the general community, hospital, clinical care centre or other health services. Exclusion criteria include individuals with overweight or obesity who do not have a diagnosis of pre-diabetes or T2DM, individuals with type 1 diabetes mellitus and individuals with conditions requiring antipsychotic drugs.

#### Intervention

Behaviour change and lifestyle interventions (e.g. with a focus on promoting physical activity and healthier eating habits) will be included, along with pharmacological treatments (e.g. lipase inhibitors such as orlistat) and nutrition therapies targeting the modification of nutrient or whole-food intake (as defined by the American Diabetes Association [27];). Pharmacological treatments must be approved as a weight loss drug by a regulatory agency (e.g. European Medicines Agency or UK Medical Medicines & Healthcare products Regulatory Agency), and food supplements to complement the normal diet must adhere to the definition provided by the Food and Drug Administration on food supplements (see: https://www.fda.gov/food/information-consumers-using-dietary-supplements/questions-and-answers-dietary-supplements). Both stand-alone and combined interventions will be included, and any delivery approach for the non-pharmacological interventions will be accepted (e.g. face-to-face, online, as part of an education programme). Papers will be excluded if the intervention focuses solely on weight loss or the weight loss component is not clearly distinguished from the weight maintenance phase. Further, studies that use Ephedra as a supplement will be excluded as it is banned in both the European Union and by the Food and Drug Administration, as well as those using Chinese traditional medicine or Ayurvedic medicine where there are no details of active ingredients/herbs utilised.

#### Control/comparator

Single-cohort studies will be accepted, as well as studies where the comparator is no intervention, standard or minimal care, waitlist or the use of a placebo. When the details of the information are unclear, authors will be contacted to provide further information.

#### Outcomes

Primary outcomes will be those most important to patients receiving the interventions. These are weight maintenance, glycaemic control (as indicated by one or more of the following: HbA1c, fasting plasma glucose, insulin sensitivity/resistance) and adverse effects. As there is no current consensus on a definition for weight maintenance, we will focus on weight difference between baseline and at least one post-intervention measurement. Adverse effects will be the physical and/or psychological side effects that may emerge from the intervention (e.g. disordered eating behaviour).

Secondary outcomes will include cardiovascular risk factors (e.g. total cholesterol, low-density lipoprotein cholesterol, high-density lipoprotein cholesterol, triacylglycerol, diastolic blood pressure, systolic blood pressure), psychological wellbeing (including health-related quality of life), change to glucose medication and waist circumference. Quantitative (both continuous and categorical) outcomes will be considered.

Should amendments be necessary, these shall be documented, reported and fully justified.

### Information sources and search strategy

A systematic search of research conducted up to the date of search initiation will be carried out. This includes searching the following databases (no restriction on publication status):

1. MEDLINE and PreMEDLINE (OvidSP) (1950 to date)
2. EMBASE Classic + EMBASE (OvidSP) (1974 to date)
3. PsycINFO (OvidSP) (1806 to date)
4. CENTRAL (The Cochrane Library)
5. ISI Web of Science: Science Citation Index Expanded (SCIEXPANDED) (1900 to date)
6. ISI Web of Science: Conference Proceedings Citation Index-Science (1990 to date)
7. International trial registers (World Health Organisation International Clinical Trials Registry portal and ClinicalStudyResults.org)

We will additionally conduct hand searches of reference lists and relevant systematic reviews, and we will review the grey literature (e.g. conference papers/conference proceedings, theses, dissertations, studies published in non-indexed journals) via OpenGrey. Personal letters and e-mails will be sent to the corresponding authors of papers in the field of weight management in T2DM. The authors will be asked for information regarding unpublished or ongoing studies.

Search terms identified for each of the following relevant categories: population (“type 2 diabetes” or “diabetes mellitus” or “diabetes mellitus type 2” or “pre-diabetes” or “prediabetes” or “hyperglycaemia”), intervention (“weight maintenance” or “maintained weight” or “weight maintenence” AND “behav*” or “lifestyle*” or “online” or “computer” or “web” or “pharma*” or “food repla*” or “*supple*”), and outcome (“weight” or “body mass index” or “BMI” or “glycaemic* control” or “cardiovascular*” or “psych*”). Boolean searches will be carried out with terms combining categories and variations of the terms (via truncation). Medical Subject Headings (MeSH) keywords and Emtree keywords will be used. The draft search strategy for MEDLINE is presented in an additional file (Additional file 2).

### Screening and data extraction

The search will be managed using the Rayyan (http://rayyan.qcri.org) software app [28]. Results will be imported into Rayyan, which automatically detects duplicates. Duplicates will also be detected through the manual data screening process. We will seek information regarding the outlined PICOs.

Two reviewers will independently screen all titles and abstracts identified by the search strategy based on the inclusion criteria. Articles that appear to meet the inclusion criteria will be extracted and reviewed in full by two authors. Inconsistencies will be discussed, and if unresolved another member of the team will be brought in to make the final decision. Studies deemed irrelevant will be excluded, and reason will be provided. Authors of unpublished studies will be contacted.

#### Semi-automated screening

Search results will additionally be uploaded to AbstrackR, a citation screening programme functioning on an algorithmic framework that predicts the likelihood citations are relevant for further inspection [29]. Screening will be initiated by two reviewers until the programme indicates it is ready to make predictions. Citations extracted through this approach will be compared and cross-checked against the output of the manual screening and selection process. Precision, false-negative rate, number of relevant citations missed and researcher time saved will be examined to determine the usefulness of AbstrackR.

#### Data extraction

Two reviewers will independently extract the data of the included studies using a standardised data extraction form shown in an additional file (Additional file 3). This includes the following:

1. Publication details (authors, title, date of publication, country of origin, funding source, corresponding author contact details)
2. Study characteristics (setting, study design, number of patients randomised to each group)
3. Participant characteristics (demographics, clinical characteristics)
4. Intervention and comparator characteristics. These will be prespecified for each type of intervention. We will also extract information from all included studies on the following factors: mode of delivery (verbal, written, computer, phone app), place of delivery, who delivered the intervention, duration of the intervention, number of sessions and format (individual or group).
5. Outcomes (as detailed in the previous section). We will record the number of participants assessed for each outcome, the mean values and standard deviations (if available) or medians and interquartile ranges for continuous data and any reported summary statistics (e.g. effect estimates, CIs, standard errors, ranges).

Inconsistencies in data extraction will be discussed and recorded. If needed, a third author will be included in the discussion to reach a final decision. When data is missing or inadequately described, three attempts will be made to request the information from the corresponding author.

### Data synthesis

A narrative synthesis of all included primary studies will be conducted. We will provide information on patient populations studied, the interventions evaluated, and any comparators used; how the studies have described and measured patient-important outcomes; and a brief summary of results in relation to weight maintenance, glucose control and adverse effects from the intervention. This will provide initial insight on study heterogeneity.

A more detailed quantitative summary of the data for each study included will be reported in a separate table. The table will show location where the study was conducted, population demographics, interventions and comparators, assessment time frame and outcomes (primary and secondary separately). An overall synthesis of the data extracted will be provided in the form of forest plots for each of the primary outcome variables. All results of both primary and secondary outcomes will be reported in a results table. Overall effect size on outcome change between baseline and follow-up assessments will be reported as weighed mean differences with 95% confidence intervals.

Meta-analyses will only be conducted if data from three or more studies were available. We will attempt to combine the results for studies that use similar interventions and report similar outcomes. However, there is likely to be heterogeneity in the types of participants, the intervention and its delivery. We will therefore be using random-effects meta-analysis models for our primary analysis to pool data across trials, where study weighing is based on in-study and between-study variances. However, fixed-effect meta-analysis models will also be explored. All meta-analyses will be carried out with the Comprehensive Meta-Analysis software [30]. Clinical heterogeneity between studies will be evaluated by identifying variability in participants, baseline data, interventions and outcomes. The I^2^ statistic will be calculated to quantify and interpret statistical heterogeneity [31]. We will apply the following thresholds for the interpretation of the *I*^2^ statistic: 0 to 40%, might not be important; 30 to 60%, may represent moderate heterogeneity; 50 to 90%, may represent substantial heterogeneity; and 75 to 100%, represents considerable heterogeneity [32].

Pooled risk ratios and 95% confidence intervals (CIs) will be calculated for dichotomous outcomes. Pooled mean differences and 95% CIs or standardised mean differences and 95% CI will be calculated for continuous outcomes when results are reported on the same scale (or can be converted to the same scale), or if results are reported on different scales, respectively. When available data does not enable statistical pooling, results will be reported in a narrative format.

#### Subgroup analyses

If there is enough data, subgroup analysis will be completed. We expect that some weight maintenance interventions may have been attached to the end of a weight loss programme, while others might be stand-alone. Factors that contribute to weight regain is time lapse since weight loss intervention [33]. Hence, effectiveness of weight maintenance interventions may be differential if they were preceded by a weight loss intervention or not. We will also compare studies based on intervention type (e.g. diet vs combined behavioural), intervention delivery format (e.g. face to face vs online) and duration of intervention, given that these characteristics have previously been linked to effectiveness in weight loss interventions [34–36].

### Missing data

If data is not available in the format required, we will first contact study authors or we will back-calculate the data if possible (e.g. standard deviation from standard errors or 95% CIs, mean and standard deviation from median and range, etc.). Reasons for missing data will be recorded (e.g. drop-outs, losses to follow-up and withdrawals).

### Risk of bias in individual studies

The Cochrane Risk of Bias Version 2 tool [37] will be used for randomised controlled trials, while the ROBINS-I tool [38] for observational studies. Two independent reviewers will assess risk of bias using the relevant tool. The latest version of the Cochrane risk of bias tool (v2 [37];) assesses each study on the following 5 domains:

- Domain 1: Risk of bias arising from the randomisation process
- Domain 2: Risk of bias due to deviations from the intended interventions
- Domain 3: Risk of bias due to missing outcome data
- Domain 4: Risk of bias in measurement of the outcome
- Domain 5: Risk of bias in selection of the reported result

Justification for decisions will be presented as supplementary material.

### Additional analyses

A sensitivity analysis will be conducted including only studies classified as low risk of bias. Any studies that had imputed results will be removed from the pooled analysis. Fixed effects meta-analyses will also be conducted. Further, we expect to carry out at least one sensitivity analysis to ensure robustness of conclusions from pooled estimate against variation in the timing of outcome assessment by eligible studies. Further sensitivity analyses may be conducted if deemed necessary depending on the studies identified from the search.

If over 10 studies are identified, a funnel plot will be generated to assess further publication bias.

### Confidence in cumulative evidence

The strength of the overall body of evidence for each outcome will be assessed using the GRADE system [39] (software: https://gradepro.org/), after generating a summary of findings table. Grading will be carried out by the team of reviewers. Grading will be based on risk of bias of individual studies, inconsistency (based on variation in point estimates, confidence intervals overlap, *p* value of heterogeneity, *I*^2^ score, outcome of the subgroup analysis), indirectness (based on differences in interventions and patients, method of measurement of patient-important outcomes and comparison of interventions), imprecision (based on Optimal Information Size, sample size, overlapping confidence intervals and judgement on the importance of observed differences) and publication bias (based on funding sources, size of studies and the comprehensiveness of data search).

## Discussion

Multiple weight loss interventions have been trialled for people with T2DM, showing substantial benefits for diabetes management [40]. Yet, weight regain shortly after the completion of a weight loss programme is common in T2DM [11]. Cost-effective interventions to prolong weight loss maintenance in T2DM are urgently needed. This review will examine the effectiveness of existing weight maintenance interventions for T2DM with the scope to inform the development of future interventions that are tailored to the needs of this clinical population. By comparing the step-by-step outputs of the systematic process when conducted solely by researchers versus when the review is supported by automated tools, we will further ascertain the amount of time saved when these tools are applied, as well as identify where human input is required to achieve accuracy. We aim to use the information to generate a standardised protocol that researchers can adhere to when employing automated tools to conduct systematic reviews on complex clinical interventions. A speedier critical appraisal of the existing literature without compromising quality will accelerate the development process of evidence-based interventions. However, a limitation of using semi-automated tools to assist screening is that these tools are highly reliant on there being enough eligible studies for “training” the system. Hence, though semi-automated tools are highly promising to facilitate reviews with large datasets, they may be less applicable with more systematic reviews targeting a less explored field of research.

We also expect that this review may require consultation with authors of candidate citations as it is common for weight maintenance interventions to be incorporated as part of a larger weight loss programme [8]. Discussions with healthcare professionals and experts in the field suggest that we will identify a limited number of studies, the interventions evaluated are likely to vary and the assessment of outcomes are likely to occur at distinct time points in relation to the intervention (particularly at follow-up). Further, behaviour change and lifestyle interventions normally address multiple factors (e.g*.* psychological, behavioural and nutritional) to enable weight maintenance. Characteristics associated with intervention effectiveness will therefore be hard to pinpoint and is likely to require in-depth analysis. Additional limitations that we may find include small sample sizes or no justification for sample size, a short follow-up in assessment and poor reporting of or no explanation for attrition. Given the above, we expect to find high heterogeneity among the included studies and the need for caution when interpreting the pooled results.

Amendments made to this protocol will be recorded on PROSPERO and reported in the final review manuscript in a table format.

## Supplementary information

**Additional file 1.** PRISMA-P 2015 Checklist. Table guiding reader to guide the reader through the sections of the protocol depicting the recommended items to be addressed by a systematic review.

**Additional file 2.** Draft Search Strategy. Document providing an example of a search strategy to be used during the database search of the systematic review.

**Additional file 3.** Data Extraction Form. Template to be used to extract relevant data of all included studies.

## Data Availability

Not applicable

## Abbreviations

T2DM: Type 2 diabetes mellitus
PRISMA-P: Preferred Reporting Items for Systematic Reviews and Meta-Analyses Protocols
PROSPERO: International Prospective Register of Systematic Reviews
PICO: Population, intervention, comparison and outcomes
CI: Confidence interval
