# Supplementary material for: Weight maintenance interventions for people with type 2 diabetes mellitus: a systematic review protocol
Source: Syst Rev. 2020 Sep 12;9:210. doi: 10.1186/s13643-020-01467-7 (PMC7488654; doi:10.1186/s13643-020-01467-7)
Supplement: Supplementary file 2 — Additional file 2. Draft Search Strategy. Document providing an example of a search strategy to be used during the database search of the systematic review. [file 13643_2020_1467_MOESM2_ESM.docx]

**Additional file 2 – Draft search strategy**

1. exp Diabetes Mellitus/di [Diagnosis]
2. (type 2 adj1 diabetes).tw
3. Diabetes Mellitus, Type 2/
4. Hyperglycemia/ or hyperglyc$emia.all
5. $diabetes.tw
6. 1 or 2 or 3 or 4 or 5
7. exp Body Weight/ or weight adj 1 maintenance.tw or weight.tw
8. (weight adj1 maint*).tw
9. (weight adj1 mana8).tw
10. maintained weight.tw
11. 7 or 8 or 9 or 10
12. Behav*.tw
13. Life style.tw or exp Life Style/
14. online.tw
15. computer.tw or Computers/
16. web.tw
17. Pharmacology/ or pharmacolog*.tw or Pharmacology, Clinical/ or exp Drug Therapy
18. (food adj1 repla*).tw
19. Supple$
20. physical activity.tw or exp Exercise/
21. 12 or 13 or 14 or 15 or 16 or 17 or 18 or 19 or 20
22. 11 and 21
23. Body Weight/ or Body-Weight Trajectory/ or weight*.tw or Body Weight Maintenance/
24. exp Body Mass Index/ or BMI
25. Glycemic Index/ or glyc$emic*.tw
26. Glycated Hemoglobin A/ or exp Blood Glucose/
27. Cardiovascular*.tw or Cardiovascular Abnormalities/ or Cardiovascular Diseases
28. Mental Health/ or Psychology/ or Mental Disorders/ or psychology*.tw
29. 13 or 24 or 25 or 26 or 27 or 28
30. Adult/
31. 6 and 21 and 29 and 30
