# Supplementary material for: Weight maintenance interventions for people with type 2 diabetes mellitus: a systematic review protocol
Source: Syst Rev. 2020 Sep 12;9:210. doi: 10.1186/s13643-020-01467-7 (PMC7488654; doi:10.1186/s13643-020-01467-7)
Supplement: Supplementary file 3 — Additional file 3. Data Extraction Form. Template to be used to extract relevant data of all included studies. [file 13643_2020_1467_MOESM3_ESM.docx]

| **Weight maintenance interventions for Type 2 Diabetes Mellitus** | | | | | |  |  |  |  |  |  |  |
| --- | --- | --- | --- | --- | --- | --- | --- | --- | --- | --- | --- | --- |
| **Patient population:** People with type 2 diabetes mellitus | | |  |  |  |  |  |  |  |  |  |  |
| **Settings:** Primary care, community, outpatient | | |  |  |  |  |  |  |  |  |  |  |
| **Intervention**: Weight maintenance | | |  |  |  |  |  |  |  |  |  |  |
| **Comparison:** No intervention, standard or minimal care, wait list, placebo and no comparator (e.g. single cohort studies) | | | | | | |  |  |  |  |  |  |
| **Outcomes:** Primary (weight, glycaemic control*^1^, adverse effects*^2^), secondary (cardiovascular risk factors*^3^, psychological wellbeing*^4^, change in glucose medication, waistline) | | | | | | | |  |  |  |  |  |
| First author, year and country | Total n of interventions | Population and clinical characteristics [mean(SD) or n (%) unless stated otherwise stated] | Demographics [mean(SD) or n (%) unless otherwise stated] | Intervention | Comparator | Primary outcomes [mean(SD) or n (%) unless otherwise stated] | Secondary outcomes [mean(SD) or n (%) unless otherwise stated] | Relative effect (95% CI, or effect size) | n of participants | Intervention fidelity: compliance, adherence | Certainty of the evidence (GRADE) | Comments |
|  |  |  |  |  |  |  |  |  |  |  |  |  |
|  |  |  |  |  |  |  |  |  |  |  |  |  |
|  |  |  |  |  |  |  |  |  |  |  |  |  |
|  |  |  |  |  |  |  |  |  |  |  |  |  |
|  |  |  |  |  |  |  |  |  |  |  |  |  |
|  |  |  |  |  |  |  |  |  |  |  |  |  |
|  |  |  |  |  |  |  |  |  |  |  |  |  |
|  |  |  |  |  |  |  |  |  |  |  |  |  |

Additional file 3 – Data Extraction Form

*^1^ Glycaemic control may be concluded from: HbA1c, fasting plasma glucose, insulin sensitivity/resistanceHbA1c, fasting plasma glucose, insulin sensitivity/resistance

*^2^ Adverse events: regain of weight lost, physical and/or psychological side effects such as disordered eating behaviour

*^3^ Cardiovascular risk factors: total cholesterol, low-density lipoprotein cholesterol, high-density lipoprotein cholesterol, triacylglycerol, diastolic blood pressure, systolic blood pressure

*^4^ Psychological wellbeing may include among others: health-related quality of life
